# Performance of Two Custom Probe Kits for In‐Solution Enrichment of Ancient Avian DNA

**DOI:** 10.1111/1755-0998.70071

**Published:** 2025-11-10

**Authors:** Chyi Yin Gwee, Laura Tassoni, Zlatozar Boev, Teresa Tomek, Zbigniew M. Bochenski, Sahra Talamo, Jochen B. W. Wolf

**Affiliations:** ^1^ Department of Evolutionary Biology LMU Munich Planegg‐Martinsried Germany; ^2^ Microevolution and Biodiversity Max Planck Institute for Biological Intelligence Seewiesen Germany; ^3^ Department of Chemistry Giacomo Ciamician Bologna University Bologna Italy; ^4^ National Museum of Natural History Bulgarian Academy of Sciences Sofia Bulgaria; ^5^ Institute of Systematics and Evolution of Animals Polish Academy of Sciences Krakow Poland

**Keywords:** birds, commercial kit, degraded, hybrid capture, target enrichment

## Abstract

Ancient DNA (aDNA) analysis remains challenging due to low endogenous DNA content of degraded samples. Hybridisation‐based in‐solution enrichment has emerged as an effective tool for targeting genomic regions, enhancing endogenous DNA yield while minimising overall sequencing effort. Despite their widespread use, the performance of different probe kits in capture efficiency remains insufficiently understood, particularly in nonhuman model organisms. In this study, we examined the performance of two commercially available custom probe systems, the RNA‐based myBaits and DNA‐based Twist, in enriching endogenous aDNA (0.9%–90.1%) extracted from crow bones collected from the early to late Holocene (100–14,000 years ago). The target regions included a panel of 104 K genome‐wide single nucleotide polymorphisms (SNPs) identified from modern populations of the *Corvus corone* species complex. Both custom probe kits substantially improved fold enrichment and target site detection rates compared with shotgun sequencing. Between the two kits, myBaits consistently achieved higher capture efficiency. In contrast, Twist retained a greater proportion of endogenous DNA, but most of this originated from off‐target regions, resulting in lower target efficiency under our experimental conditions. Twist demonstrated higher coverage in regions with extreme GC content, highlighting its utility for applications targeting GC‐rich genomic regions. These findings provide insights into the performance of commercially available DNA enrichment methods and help guide study design.

## 1 Introduction

Over the past decade, next‐generation sequencing (NGS) has become a cornerstone of genomics, enabling rapid, high‐throughput sequencing at reduced costs. Despite these advancements, analysing ancient DNA (aDNA) remains challenging due to the low amount of endogenous DNA, necessitating deep sequencing (Carpenter et al. 2013). In‐solution enrichment strategies using hybridisation‐based probes overcome this limitation by efficiently capturing target regions, increasing the depth of endogenous DNA while reducing the overall sequencing depth (Gnirke et al. 2009). This approach has proven transformative for researchers working with degraded samples from historical and ancient specimens.

Initially, in‐solution enrichment methods were limited to laboratories capable of synthesising custom probes, derived from sources such as microarrays (Fu et al. 2013; Gnirke et al. 2009), PCR products (Horn 2012; Maricic et al. 2010; Noonan et al. 2006; Reich et al. 2010; Sawyer et al. 2012; Schuenemann et al. 2011) or modern DNA libraries (Carpenter et al. 2013). However, since the early 2010s, the availability of commercial kits has made this technology more broadly accessible, facilitating aDNA studies in a wide range of organisms, including birds (Mitchell et al. 2014), plants (Ávila‐Arcos et al. 2011; da Fonseca et al. 2015; Mascher et al. 2016), mammals (Enk et al. 2013; Kihana et al. 2013; Paijmans et al. 2016; Vilstrup et al. 2013), viruses (Duggan et al. 2016; Mühlemann et al. 2018) and bacteria (Andrades Valtueña et al. 2017; Bouwman et al. 2012; Furtwängler et al. 2020; Maixner et al. 2016; Wagner et al. 2014).

While these kits are based on similar principles of using biotinylated probes to capture and isolate target DNA regions, they differ in the type of nucleic acid used. For instance, SureSelect (Agilent Technologies) and myBaits (Arbor Biosciences, formerly MYcroarray) utilise single‐stranded RNA (ssRNA) probes, while Roche's NimbleGen SeqCap EZ system, its updated version KAPA HyperChoice and xGen Custom Hyb Panels (Integrated DNA Technologies) utilise single‐stranded DNA (ssDNA) probes. More recently, Twist Bioscience has introduced customisable double‐stranded DNA (dsDNA) probes into the market.

Although in‐solution enrichment strategies are widely applied to studies involving degraded DNA (Gasc et al. 2016; Lan and Lindqvist 2018), there remains a significant gap in understanding how different probe types may influence aDNA capture efficiency, especially in nonmodel organisms. RNA probes may form more stable RNA–DNA hybrid duplexes (Chein and Davidson 1978; Lesnik and Freier 1995), but comparative studies yield varying conclusions with the application of different commercially available probes: Some report no significant performance difference (Sulonen et al. 2011), others suggest higher capture rates for RNA probes (Zhou et al. 2021) and others found better performance by DNA probes (Bodi et al. 2013; Rohland et al. 2022). Even less is known about how probe performance may vary across AT‐ and GC‐rich regions of the genome (Zhou et al. 2021).

Additionally, the reagents and protocols provided by different commercial kits play a significant role in probe performance. These kits are frequently updated by manufacturers to enhance target specificity, as reflected in the release of new protocols and reagent versions. While users can optimise certain aspects of the protocol, such as incubation time and temperature (Cruz‐Dávalos et al. 2017; Paijmans et al. 2016), these adjustments are typically constrained by the standardised reagents and procedures provided, in addition to the cost in terms of time and effort. Moreover, many users lack the specialised knowledge or resources to modify the reagents or fundamentally alter the standard protocols. Therefore, the enrichment efficiency largely depends on the quality of reagents and the robustness of the protocols developed by each manufacturer.

In this study, we compared the performance of two commercially available probe kits, the RNA‐based myBaits and DNA‐based Twist. Following the standard protocols provided by each manufacturer, we evaluated their effectiveness in enriching aDNA libraries generated from 23 crow bones spanning 100 to 14,000 years of age. The custom panel includes 104 K genome‐wide single nucleotide polymorphisms (SNPs) identified from modern populations of the *Corvus corone* species complex (Poelstra et al. 2014; Vijay et al. 2016). Our findings contribute to a growing understanding of commercially available probe performance in aDNA studies, offering insights into the suitability of different systems for enriching degraded DNA in an avian species.

## 2 Materials and Methods

### 2.1 Sample Collection and Radiocarbon Dating

A total of 23 bone samples from hooded crows were collected from Poland (*n* = 13) and Bulgaria (*n* = 10; Table S1). Subsamples weighing 40–274 mg were taken for collagen extraction pretreatment (Fewlass et al. 2019) and the graphitisation process (Tassoni et al. 2023) at the Bologna Radiocarbon laboratory devoted to Human Evolution (BRAVHO). Accelerator Mass Spectrometry (AMS) dating was carried out at the Curt‐Engelhorn‐Center Archaeometry (CEZA) in Mannheim, Germany. The radiocarbon (^14^C) ages of the samples were inferred to range between 164 ± 18 BP and 13,732 ± 37 BP (Table S2).

### 2.2 DNA Extraction and Library Preparation

A total of 20 mg bone powder was obtained from each sample in a clean room at the Max Planck Institute for Evolutionary Anthropology (MPI‐EVA) in Jena. To minimise exogenous contamination, the inner surface layer of each hollow avian bone was lightly scrapped with a Dremel tool. The outermost layer of bone powder generated during this initial cleaning was discarded to ensure any potential contaminants were removed. Subsequently, bone powder from the underlying layer, which appeared visibly whiter, was collected for DNA extraction. Extraction of aDNA from bone powder was conducted following an established pipeline (Rohland et al. 2018) and single‐stranded DNA (ssDNA) libraries were generated without UDG treatment by automated machines at MPI‐EVA (Gansauge et al. 2017, 2020; Nagel et al. 2024). Each library was amplified and indexed via PCR with a unique set of customised dual indexes to allow multiplex sequencing. The final libraries were therefore double‐stranded before being shotgun sequenced at shallow levels on the Illumina HiSeq 2000 platform at MPI‐EVA to assess the quality and quantity of endogenous DNA of each library.

### 2.3 SNP Panel Design

**FIGURE 1:**
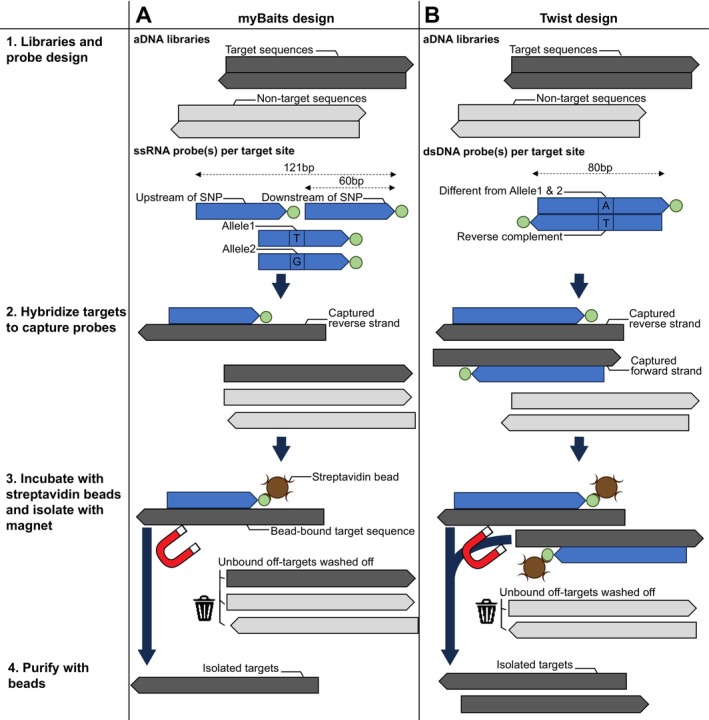
Schematic representation of the (A) myBaits and (B) Twist design for in‐solution enrichment of endogenous crow DNA libraries. Legend: The dark and light grey strands represent on‐target and off‐target sequences, respectively. The blue strands represent biotinylated probes with biotin attached to the 3′ end (green dot). The biotinylated probes are single‐stranded RNA (ssRNA) for myBaits and double‐stranded DNA (dsDNA) for Twist. Arrows indicate 5′‐3′ directionality.

Two commercial kits, myBaits Custom 380–400 K DNA‐seq version 5 (Daicel Arbor Biosciences, Michigan, USA) and Twist Custom Panel (Twist Bioscience, South San Francisco, USA) were utilised to enrich for 103,769 SNPs and 232,015 SNPs, respectively (Figure 1). The myBaits 104 K panel was designed to target each SNP using four 60 bp single‐stranded RNA (ssRNA) probes, such that one probe aligns upstream just before the SNP, one probe aligns downstream just after the SNP, and two probes overlap the SNP itself, each representing one of the two known alleles (Fu et al. 2016; Haak et al. 2015). The Twist 232 K panel encompasses all SNPs from the above 104 K panel, with additional SNPs from the inclusion of one more discovery population (see Data S1 for details on SNP selection). Each SNP is targeted by a single 80 bp double‐stranded DNA (dsDNA) probe. These probes are designed to overlap the SNP site, with each probe containing a nucleotide different from both known alleles to prevent ascertainment bias (Rohland et al. 2022). Overall, the mean GC content of the 104 and 232 K probes is 45.8% and 41.8%, respectively, which is similar to the GC content of the hooded crow reference genome (GenBank accession no. GCA_000738735.1; GC = 41.5%) and falls within the commercially recommended range for probe design (between 30% and 70%) to reduce capture biases and off‐target binding.

### 2.4 In‐Solution Hybridisation

A total of 23 indexed double‐stranded DNA libraries prepared with ssDNA protocol (see description for library preparation above) were enriched separately using myBaits and Twist probes, following the High Sensitivity Protocol v.5.03 and Target Enrichment for Ancient DNA Rev4 protocol provided by each manufacturer, respectively. Each library underwent a single round of enrichment. A summary of the standard protocols is provided below, with all modifications explicitly stated:

For the myBaits enrichment, the Hybridization Mix was prepared following the protocol, using four Hyb reagents and 5.5 μL of probes. The Hybridization Mix was incubated at 60°C for 10 min, after which 18.5 μL was aliquoted into each well of a strip tube to form the ‘HYBs’. The Blockers Mix was prepared, following the protocol for most taxa, using Block O, Block C and Block X, and 7 μL of each ssDNA library was added to form the ‘LIBs’. The LIBs were put in a thermal cycler at 95°C for 5 min followed by incubation at 60°C for another 5 min, jointly with the tubes containing HYBs added to the thermal cycler. Afterward, 18 μL of each HYB was pipetted into each LIB, mixed by pipetting and incubated at 60°C for 24 h with the lid maintained at 65°C. For each capture reaction, 30 μL of Streptavidin‐coated beads (included in the kit) were washed three times with 200 μL of Binding Buffer, resuspended in 70 μL of Binding Buffer and heated at 60°C for 2 min. After hybridisation, the DNA‐probe complexes were transferred to 70 μL of the resuspended beads, incubated at 60°C for 5 min, and washed on the thermal cycler at 60°C four times with 180 μL of freshly prepared Wash Buffer X to remove any unbound DNA. After the last wash and pelleting, the beads were resuspended in 30 μL of Buffer E and mixed by pipetting before proceeding to amplification.

For the Twist enrichment, 5 μL of Hybridization Mix (Hyb reagents already assembled by the manufacturer) was added to 1 μL of probes to form the ‘HYBs’. The Blockers Mix was prepared with 5 μL of Blocker Solution and 7 μL of Universal Blockers, combined with 5 μL of each ssDNA library (instead of the dried pellet specified in the protocol) to form the ‘LIBs’. The HYBs were put in the thermal cycler at 95°C for 2 min, then cooled on ice for 5 min. Simultaneously, the LIBs were put in the thermal cycler at 95°C for 5 min, then both HYBs and LIBs were left at room temperature for 5 min. Each HYB was added to a LIB, mixed by pipetting, and added with 30 μL of Hybridization Enhancer to the top of the reaction. The reactions were incubated at 62°C for 16 h with the lid maintained at 65°C. For each capture reaction, 300 μL of Streptavidin‐coated beads (included in the kit) were washed three times with 600 μL of Binding Buffer, resuspended in 200 μL of Binding Buffer, and heated at 62°C for 10 min. After hybridisation, the DNA‐probe complexes were transferred to 200 μL of the resuspended beads on the thermal cycler at 62°C, mixed by pipetting, removed from the thermal cycler, and incubated at room temperature for 30 min. Bead washing was performed at the bench: one wash with 200 μL of Wash Buffer 1, followed by three washes with 200 μL of Wash Buffer 2, with each wash incubated at 46°C for 5 min (the temperature was reduced from a default of 48°C as recommended by the manufacturer to reduce AT dropout).

Both myBaits‐ and Twist‐enriched libraries were amplified in 15 cycles of PCR using the Equinox Library Amplification Kit (Watchmaker Genomics, Colorado, USA) with i5 and i7 primers. The number of PCR cycles was optimised to 15 after experimenting with 23 and 21 cycles, which resulted in the appearance of a second, larger peak on the bioanalyzer. This peak indicated the formation of heteroduplex bubble products, a common artifact of excessive PCR cycling. Following PCR, the libraries were cleaned using the DNA Purification Beads provided by the Twist kit. The final concentration of each enriched library was quantified using an Invitrogen Qubit 3.0 Fluorometer.

### 2.5 Sequencing

A total of 46 enriched libraries and four nonenriched whole genome libraries (BRW001, DVT014, NCP002 and VKP001) were sequenced on the Illumina NovaSeq 6000 platform using 75 bp paired‐end reads. Sequencing was carried out across two flow cells comprising eight lanes, which also included other ancient libraries unrelated to this study. Libraries prepared by each enrichment approach were pooled by equimolar amounts and sequenced with approximately 1.4% of a lane allocated per sample, ensuring that the sequencing effort was distributed as evenly as possible across enriched libraries. Although some variability in sequencing depth is inevitable, the average ratio of raw reads generated per sample across both enrichment approaches was 1.01, indicating that the sequencing effort was closely matched. The nonenriched libraries were sequenced with a higher effort, utilising approximately 40% of a lane per sample. One exception was sample BRW001, which exhibited high endogenous DNA content (90.1%) during the shallow shotgun sequencing at MPI‐EVA; this sample was sequenced with twice the effort (i.e., 80% of a lane) to maximise coverage.

### 2.6 Bioinformatic Raw Data Processing

The sequence reads generated from the two enrichment approaches and shotgun sequencing (for samples BRW001, DVT014, NCP002 and VKP001) were processed independently using nf‐core/eager 2.4.7, a reproducible pipeline for the analysis of degraded DNA (Fellows Yates et al. 2021). The pipeline workflow is divided into two parts: one for raw sequence data and another for processing BAM files.

In the first part of the pipeline, AdapterRemoval (Schubert et al. 2016) was used to identify and trim adapter sequences. A list of seven adapter pairs, accessible on our GitHub depository (https://github.com/EvoBioWolf/CORVID_aDNAenrichment), was provided to trim the customised adapters used for ssDNA library preparation and two pairs of over‐represented sequences flagged by FastQC (Andrews 2010).

The adapter‐trimmed reads were mapped to the hooded crow reference genome (GenBank accession no. GCA_000738735.1) with chromosome W (Warmuth et al. 2022) manually added to the FASTA file. Mapping was performed using BWA 0.7.17 (Li and Durbin 2009) with the following parameters: bwa aln ‐l 1024 ‐n 0.04 ‐o 2. Seeding was disabled (−l 1024) to enable alignment of the entire read, rather than relying on seeds. While seeding accelerates the alignment process, it can result in missed alignments in aDNA due to mismatches caused by deamination pattern and multiple alignment with short seed lengths (Schubert et al. 2012). Therefore, alignment and mapping were executed outside the nf‐core pipeline to allocate additional memory and CPU parallelisation to this step. The resulting SAM files were converted to BAM format using SAMtools 1.7 (Li et al. 2009) without additional quality filtering.

In the second part of the pipeline, the BAM files underwent several post‐processing steps, including duplicate removal, deamination rate calculations and trimming of 5 bp from both ends of each mapped read. A MultiQC (Ewels et al. 2016) report was generated at the end to summarise various metrics for each enriched library sample. For all enriched libraries, we generated reports to obtain the mean coverage and number of unique reads aligning to (1) the 80 bp bait length of the 104 K panel (comparable to both enrichment systems), (2) the SNP sites of the 104 K panel (comparable to both systems), (3) the 121 bp bait length of the original 104 K panel of myBaits system, (4) the SNP sites of the 232 K Twist panel and (5) the full 80 bp bait length of the 232 K panel of the Twist system.

### 2.7 On‐Target Alignment

To compare the efficiency of target enrichment across methods, we defined ‘comparable targets’ as the intersection of the 104 K 121 bp myBaits panel with the 232 K 80 bp Twist panel (i.e., the 80 bp target regions of the 104 K panel shared by both methods). Genomic regions uniquely captured by a single method (referred to as ‘noncomparable targets’) were excluded from comparative analyses. To evaluate whether performance assessments differed when using the full probe designs, we also calculated enrichment efficiency using the original panels: the 104 K panel with 121 bp probes for myBaits and the 232 K panels with 80 bp probes for Twist. Below, we describe the two metrics used to assess enrichment efficiency, using the comparable‐target approach as an example:

On‐target rate was calculated as the proportion of reads mapped to comparable targets, including duplicates, relative to the total number of raw reads, excluding those mapped to noncomparable targets. This metric reflects the proportion of on‐target alignment rate relative to all sequenced data, including any environmental contaminants and adapter sequences:

$$\mathrm{On}-\text{target rate}=\frac{\text{Reads mapped to comparable targets}}{\left(\text{Total}\;\mathrm{raw}\;\text{reads}\right)-\left(\text{Reads mapped to noncomparable targets}\right)\;}\times 100$$

Target efficiency was calculated as the proportion of reads mapped to comparable targets, including duplicates, relative to the total number of reads mapped to the hooded crow genome, excluding noncomparable targets. Unlike on‐target rates, this metric accounts only for endogenous mapped reads, reducing noise from off‐content regions deriving from exogenous DNA and unremoved adapter sequences. The formula is as follows:

$$\text{Target efficiency}=\frac{\text{Reads mapped to comparable targets}}{\left(\text{Total mapped reads}\right)-\left(\text{Reads mapped to noncomparable targets}\right)\;}\times 100$$

### 2.8 Fold Enrichment

To directly assess the fold enrichment achieved by each method, we compared the proportion of target sequences before and after in‐solution hybridisation for four shotgun‐sequenced samples (BRW001, DVT014, NCP002 and VKP001). Specifically, the target efficiency (see formula above) calculated after enrichment was compared with that obtained from the corresponding shotgun sequencing of each sample. This comparison was performed using both the comparable and original panels.

### 2.9 Coverage of Target Sites

The observed coverage of each enriched library was calculated as the average number of reads mapped to the 104 K target SNP sites. Coverage evenness was assessed by calculating the proportion of SNPs in each sample reaching a minimum threshold of 1×–5× across both methods, along with the total number of samples in which at least 80% of SNPs meet the respective threshold.

Additionally, as background information, the expected genomic coverage for each DNA library was estimated using an indirect approach described by Gansauge et al. (2020). This method calculates the total cumulative length of endogenous DNA fragments in a library, referred to as informative sequence content, and integrates data from shallow shotgun sequencing to measure both the amount of endogenous DNA and the mean mapped read length, as well as the total number of DNA molecules (endogenous and nonendogenous) quantified by qPCR (qPCR mol; see Table S3). It assumes that reads are uniformly distributed across the genome and there are minimal sequencing duplicates, such that the informative sequence content can be divided by the total genome size to estimate expected genomic coverage. The expected coverage of each library (eluted in 50 μL) was calculated using the following formula:

$$\text{Expected coverage of library}=\frac{\text{qPCR}\;\mathrm{mol}\times \frac{\mathrm{All}\;\text{mapped reads}}{\text{Total reads}}\times \text{Mean read length}}{\text{Total hooded crow genome length}\;\left(=1.12\;\text{billion}\;\mathrm{bp}\right)}$$

Since 7 and 5 μL of the DNA libraries were used for myBaits and Twist, respectively, the expected coverage of each capture library was adjusted accordingly: 7/50 of the total library coverage for myBaits and 5/50 for Twist.

### 2.10 Coverage Across Varying GC Content

To assess GC bias in each sample, we ran CollectGcBiasMetrics (http://broadinstitute.github.io/picard), which evaluates the proportion of guanine (G) and cytosine (C) nucleotides across the target regions of each sample. For each sample from each enrichment approach, a detailed metrics table was generated. This table includes: (i) number of 80 bp windows in the reference probes assigned to each GC bin, (ii) mapped read count for each GC bin, representing reads aligned to a window with a specific GC content, and (iii) normalised coverage for each bin, calculated as the ratio of the bin's coverage relative to the mean coverage across all GC bins (i.e., normalised coverage of 1 represents the mean).

Additionally, summary metrics were generated for each sample, including AT and GC dropout. These metrics calculate GC bias by comparing GC content distribution between reference windows and mapped reads bins. AT dropout is the sum of positive differences between the percentages of reference windows and mapped reads for GC bins from 0 to 50. Similarly, GC dropout is the sum of positive differences for GC bins from 50 to 100. This approach provides a comprehensive view of GC bias across different enrichment methods, allowing for the comparison of coverage uniformity across samples.

### 2.11 DNA Damage Pattern of Captured Reads

Ancient and degraded DNA is characterised by the deamination of cytosine residues to uracil, which are read as thymine during sequencing (Briggs et al. 2007). To examine deamination patterns in each sequenced library, we assessed the percentage of reads showing C→T substitutions at the 5′ end of sequences.

### 2.12 Genotyping and Allele Bias

To assess potential capture bias at heterozygous SNP sites from the 104 K panel, we generated whole‐genome shotgun libraries for four samples (BRW001, DVT014, NCP002, and VKP001) which were processed with the nf‐core/eager 2.4.7 pipeline as described above. Genotype calling was conducted on both the shotgun‐sequenced and enriched datasets using ANGSD 0.935 (Korneliussen et al. 2014) with the following parameters: angsd ‐bam bam.filelist ‐GL 2 ‐doMaf 2 ‐doMajorMinor 1 ‐doGeno 4 ‐doPost 1 ‐doGlf 2 ‐doCounts 1 ‐doPlink 2 ‐minMapQ 20 ‐minQ 20 ‐uniqueOnly 1 ‐remove_bads 1 ‐geno_minDepth 3 ‐nThreads 8 ‐rf probes_104k_SNPsite.1based.

To evaluate genotyping accuracy of the enrichment methods, genotypes from each method were compared to those from shotgun sequencing, which served as the baseline. Given the potential for low‐coverage shotgun sequencing to miss sampling of the alternative allele by chance, a genotype was considered a match if at least one of the two alleles identified in the enriched data set was also present in the baseline.

To investigate allelic bias, we focused on biallelic heterozygous sites present in the shotgun data and both enrichment data sets with a minimum genotype depth of 3×. For each of the six possible biallelic variant classes (AG, CT, AC, AT, CG and GT), allele depths were calculated separately for the shotgun, myBaits and Twist data sets. Read counts for each allele at each variant site were computed using ANGSD with the following parameters: angsd ‐doCounts 1 ‐dumpCounts 4 ‐bam bam.filelist ‐minMapQ 20 ‐minQ 20 ‐uniqueOnly 1 ‐remove_bads 1 ‐rf probes_104k_SNPsite.1based.

Allelic bias was statistically assessed using a quasibinomial generalised linear model (GLM) in R 4.3.3 (see R script at https://github.com/EvoBioWolf/CORVID_aDNAenrichment). The model treats the counts of the two alleles at each variant site as a binomial outcome with an expected probability of 0.5 for drawing either allele. Allelic bias was inferred when the estimated allele depth ratio significantly deviated from 0.5, using a *p* value threshold of 0.05. A Bonferroni correction was applied within each sample to adjust for multiple testing (adjusted significance threshold: *p* < 0.05/18). The distribution of allele depth ratios across all samples and variant classes was visualised with violin plot on R.

## 3 Results

### 3.1 Feature Comparison

The myBaits system utilises four 60 bp RNA probes per SNP, covering a total length of 121 bp, including the SNP position. This allows for up to 4× probe coverage on the minus strand (Figure 1). In contrast, the Twist system employs a single 80 bp DNA probe, covering a total length of 80 bp, including the SNP position, and enables up to single probe coverage, yet on both the plus and minus strands. These system differences highlight contrasting strategies in coverage and strand specificity.

### 3.2 Capture Efficiency

**FIGURE 2:**
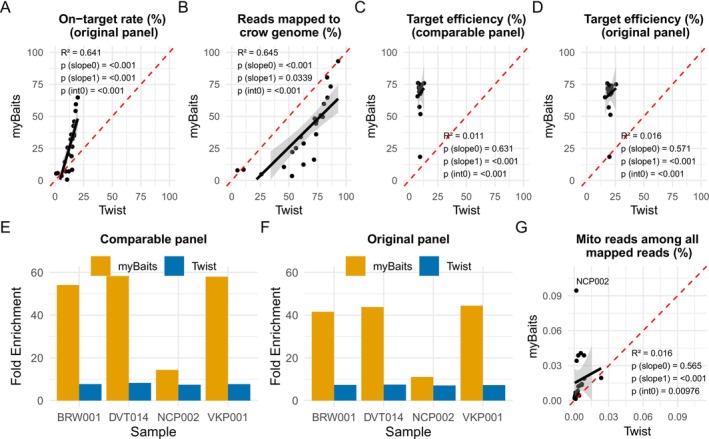
Comparisons of capture efficiency between myBaits and Twist. Each dot represents one of 23 samples for which both capture methods were applied for plots A–D and G. The red dashed line indicates the expected 1:1 relationship under equal performance, the black solid line shows the observed linear regression, and the grey shaded area depicts the 95% confidence interval of the regression. Correlation value (*R* ^2^), the significance of the linear relationship (*p*(slope0)), the significance of nonproportional scaling (*p*(slope(1))) and the significance of a nonzero intercept (*p*(int0)) are reported in each plot. (A) On‐target rate measured as the percentage of on‐target reads relative to all raw reads (including unmapped reads from exogenous DNA). (B) Percentage of all raw reads (including duplicates) mapped to the hooded crow reference genome. This includes both on‐target and off‐target reads and thus does not directly reflect target enrichment. (C) Target efficiency measured as the percentage of on‐target reads aligning to the comparable 104 K SNP panel with 80 bp probes relative to all reads mapped to the hooded crow genome. (D) Target efficiency measured as the percentage of on‐target reads aligning to the original panel: 104 K SNP panel with 121 bp probes for myBaits and 232 K SNP panel with 80 bp probes for Twist, relative to all mapped. (E) Fold enrichment for four samples before and after capture, based on the comparable panel. The endogenous DNA content of the samples from left to right: 90.1%, 19.5%, 3.1% and 24.7% (by shotgun sequencing). (F) Fold enrichment based on the original panel. (G) Percentage of mitochondrial reads among all reads mapped to the hooded crow genome.

Overall, the myBaits system exhibited a higher on‐target rate than Twist for both the original and comparable panels (mean 26.8% for myBaits vs. 12.9% for Twist in the original panel, and 26.9% for myBaits vs. 5.88% for Twist in the comparable panel; Figure 2A, Table S4). As on‐target rates can be influenced by the amount of exogenous DNA and residual sequencing adapters, metrics (e.g., target efficiency and fold enrichment) specific to reads mapped to the hooded crow genome may be more informative. Twist retained a higher proportion of endogenous DNA than myBaits (mean 63.6% vs. 37.8% including duplicates, and 17.6% vs. 7.8% after duplicate removal; Figure 2B, Table S4). However, most of this endogenous DNA corresponded to off‐target regions, resulting in lower overall enrichment efficiency. In contrast, myBaits exhibited substantially higher target efficiency relative to both the original panel design and comparable 104 K panel with 80 bp probes (Figure 2C,D, Table S4). Mean target efficiency for myBaits ranged from 68.2% to 68.6%, compared to 9.6% to 20.2% for Twist and 54.4% to 54.6% vs. 10.8% to 22.1% after duplicate removal.

To directly compare the panel, we excluded targets unique to either approach from both the on‐target count (nominator) and the total mapped reads (denominator). After adjusting for the 128 K additional sites unique to Twist's 232 K panel, the target efficiency of Twist for the comparable 104 K panel was approximately halved (9.6% vs. 20.2%). In contrast, adjusting for the longer probe length of myBaits (from 121 bp to 80 bp) had minimal effect on its target efficiency (68.2% vs. 68.6%). We tested additional adjustments by reducing the proportion of off‐target reads to reflect the potential for increased off‐target capture in a larger panel, and increasing the on‐target reads for Twist by 2× to approximate the higher probe coverage of myBaits. However, in all scenarios, myBaits consistently exhibited higher target efficiency (Figure S1). Taken together, these results demonstrate that myBaits achieved greater capture enrichment efficiency under our experimental conditions.

To directly quantify the effectiveness of target enrichment, we calculated fold enrichment for four samples that were also shotgun sequenced, allowing a direct comparison of target efficiency between capture methods and shotgun sequencing. Both kits substantially enriched the target regions, but with clear differences: myBaits achieved an average fold enrichment of 35.2×–46.2×, while Twist achieved 7.28×–7.78× (Figure 2E,F). Fold enrichment was consistently higher in myBaits across all samples. Specifically, sample NCP002, which had the lowest endogenous DNA content (3.1%), showed lower fold enrichment by myBaits (11.0×–14.4×) than other samples. In contrast, Twist consistently showed a fold enrichment of ~8× across all samples, even when the endogenous DNA content was as high as 90.1% in sample BRW001. A similar limiting trend was observed in the narrow range of target efficiencies across samples of varying endogenous content in Twist compared with the broader efficiency range in myBaits (Figures 2B,C and S2A–D), suggesting that the Twist enrichment may have reached a saturation point independent of the endogenous DNA content in our experiment.

Despite its higher efficiency, myBaits exhibited a greater proportion of mitochondrial reads among all mapped reads, even though mitochondria were excluded from the panel to prevent over‐enrichment (Figures 2G and S2E,F). Overall, both designs captured a very low number and proportion of mitochondrial reads (0.0173% for myBaits vs. 0.00434% for Twist; Figures 2G and S2E,F, Table S4), suggesting that co‐capture due to sequence similarity is unlikely. The consistently low proportion of mitochondrial reads in Twist libraries further suggests that the off‐target endogenous DNA reads in Twist primarily originate from the nuclear genome. For the poor‐quality sample with low endogenous DNA content (NCP002), mitochondrial reads exceeded 0.09% of mapped reads in the myBaits enriched library, compared to only 0.0018% in the Twist‐enriched library, further suggesting a higher affinity of myBaits for mitochondrial DNA fragments.

### 3.3 Target Coverage and Evenness

**FIGURE 3:**
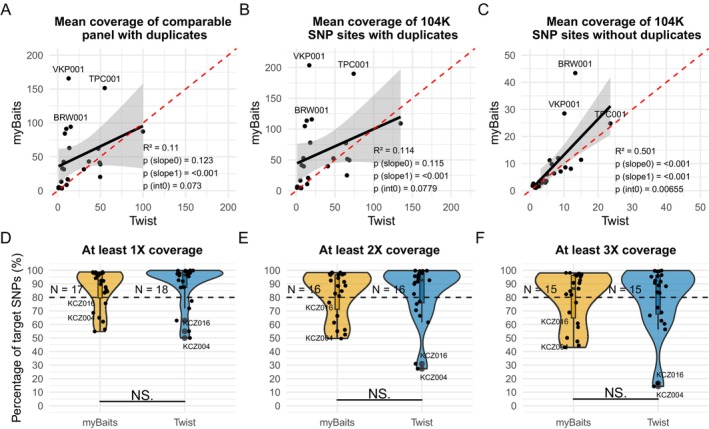
Comparisons of coverage across target sites and enrichment evenness between myBaits and Twist. Each dot represents one of 23 samples to which both strategies were applied. For plots (A–C), the red dashed line indicates the expected 1:1 relationship under equal performance, the black solid line shows the observed linear regression, and the grey shaded area depicts the 95% confidence interval of the regression. Correlation value (*R* ^2^), the significance of the linear relationship (*p*(slope0)), the significance of nonproportional scaling (*p*(slope(1))), and the significance of a nonzero intercept (p(int0)) are reported in each plot. (A) Mean coverage of the comparable panel consisting of 104 K SNPs with 80 bp probes, with duplicated reads included. Samples with the three highest levels of endogenous DNA (BRW001, VKP001, and TPC001) are labelled. (B) Mean coverage of the 104 K SNP sites with duplicated reads included. (C) Mean coverage of the 104 K SNP sites with duplicated reads removed. (D–F) Enrichment evenness assessed as the percentage of SNPs reaching minimum coverage thresholds of 1×, 2× and 3×, respectively. The number of samples with at least 80% of SNPs meeting each minimum coverage threshold is indicated as ‘*N* = X’ next to each boxplot. Two outlier samples (KCZ004 and KCZ016) are labelled next to their respective data points.

Following the assessment of overall capture efficiency, we next quantified coverage distribution across the target regions (Figure 3A) and SNPs (Figure 3B,C). Overall, myBaits achieved higher mean coverage than Twist across both the comparable target panel (49.4× vs. 23.3× including duplicates; Figure 3A) and the 104 K SNP sites (56.1× vs. 33.6× with duplicates, and 8.9× vs. 6.7× without duplicates; Figures 3B,C and S2G,H, Table S4). However, in contrast to the overall target efficiency (Figure 2C,D) most samples showed comparable coverage between the two methods after duplicate removal, indicating unique molecules covering these target sites were similarly captured by both methods (Figure 3C, Table S5).

To assess the homogeneity of capture efficiency across target sites, we calculated the percentages of SNPs in each sample reaching minimum coverage thresholds of 1×, 2× and 3×, and counted the number of samples in which at least 80% of SNPs met each coverage threshold (Figure 3D–F; see Table S4 for mean percentages of SNPs covered at 1×–5× with and without duplicates). Overall, the enrichment distributions were comparable between the two methods. However, Twist slightly outperformed myBaits in the number of samples achieving at least 80% of SNP coverage at the 1× threshold and displayed a narrower coverage distribution across SNPs, with the exception of two samples (KCZ004 and KCZ016), which consistently showed poorer SNP coverage in Twist compared to myBaits.

### 3.4 Coverage Across GC Content

**FIGURE 4:**
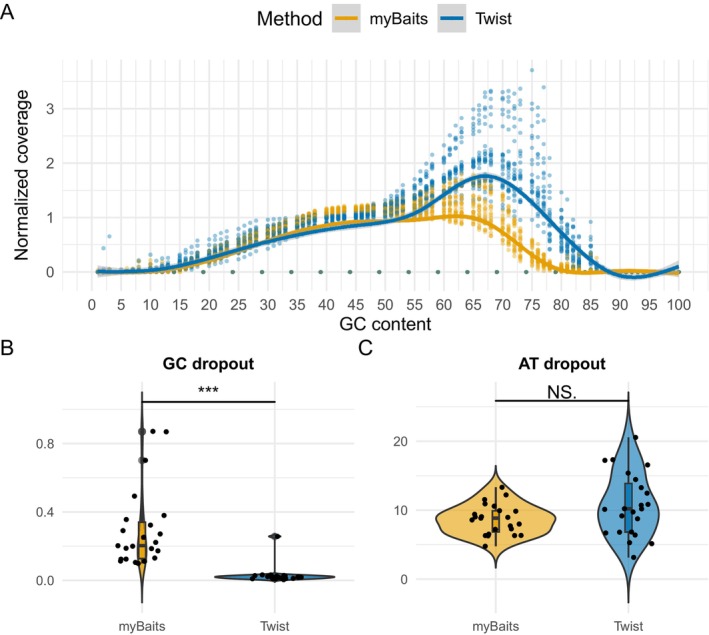
Enrichment efficiency at different GC content. (A) Normalised coverage across GC content of reads mapped to 104 K SNP panel of 80 bp. (B) Percentage of GC dropout at regions with high GC content (GC > 50). (C) Percentage of AT dropout at regions with low GC content (GC < 50).

Normalised coverage across GC content for reads mapped to the 104 K SNP panel was similar between myBaits and Twist in regions with low‐to‐moderate GC content (GC < 50), corresponding to comparable AT dropout rates between the two systems (Figure 4A,C). However, significant differences emerged at higher GC content. Twist showed higher normalised coverage (> 1×) in regions with GC content between 55% and 80%. Normalised coverage decreased rapidly at GC content above 80%, indicating reduced efficiency at extreme GC content (Figure 4A,B). In comparison, myBaits maintained near‐normalised coverage in regions with GC content between 55% and 70%, but performance dropped below one at GC levels above 70%, indicating that myBaits was less efficient than Twist in regions with extreme GC content.

### 3.5 Read Length and Postmortem DNA Damage Patterns

**FIGURE 5:**
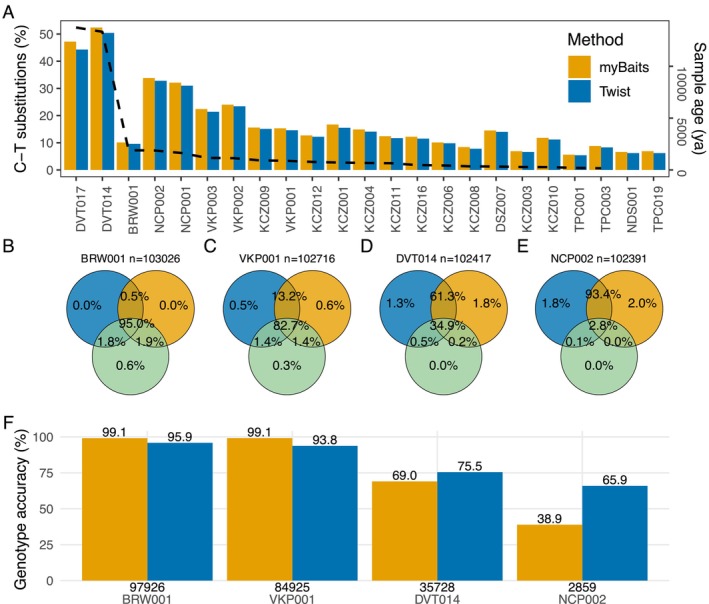
SNP sites detection and genotyping accuracy. (A) C to T substitution rates on the first base pair (bp) of each read indicating postmortem DNA damage covary‐with the corresponding age of the sample (dashed line). (B–E) Total number of SNPs from the 104 K SNP panel discovered across the same four individuals using myBaits (orange), Twist (blue), and shotgun‐sequencing (green). Venn diagram of samples arranged in descending order of endogenous DNA content. (B) Sample BRW001 with 90.1% endogenous DNA and shotgun sequence to 43.4× (without duplicates). (C) Sample VKP001 with 24.7% endogenous DNA and shotgun sequence to 7.5×. (D) Sample DVT014 with 19.5% endogenous DNA and shotgun sequence to 4.4×. (E) Sample NCP002 with 3.1% endogenous DNA and shotgun sequence to 0.9×. (F) Genotype accuracy of each sample captured by myBaits and Twist against genotype identified by shotgun sequencing. The number of overlapping sites, including homozygous genotype, used in the comparison is shown at the bottom of each bar plot for each sample.

Read length and percentage of C→T substitution on the start of each on‐target read were highly comparable across both systems (Table S4), though myBaits showed slightly higher C→T deamination than Twist in the enriched on‐target reads, particularly for older samples (> 10,000 years; Figure 5A). This pattern suggests that myBaits might be slightly more effective at capturing degraded reads with high deamination rates, potentially offering a slight advantage in recovering damaged ancient DNA fragments.

### 3.6 Genotyping and Allele Bias

Both methods detected a similar number of SNP sites from the 104 K panel and significantly outperformed shotgun sequencing in terms of target site recovery (Figure 5B–E). In the high‐quality sample (BRW001), up to 95% of recovered sites were shared across all three methods (Figure 5B). In contrast, in the low‐quality sample (NCP002), more than 90% of recovered sites were unique to the enriched libraries and not detected in the shotgun data (Figure 5E). This demonstrates the effectiveness of target enrichment in retrieving SNPs from degraded samples, where shotgun sequencing alone would have limited genotyping success. Notably, the sequencing depth for enriched libraries was approximately 1/40th of that for shotgun libraries and 1/80th for BRW001, underscoring the cost‐efficiency of enrichment approaches.

Both enrichment methods also showed comparable genotyping accuracy, with myBaits demonstrating slightly higher accuracy in better‐quality samples (BRW001 and VKP001), and Twist achieved higher accuracy in the more degraded samples (DVT014 and NCP002; Figure 5F). The lower genotyping accuracy observed in degraded samples is likely due to issues with read quality rather than inherent differences in enrichment accuracy. Importantly, both kits achieved high genotype concordance with the shotgun sequencing baseline in good‐quality samples, with allele matches reaching up to 99.1% for myBaits and 95.9% for Twist, indicating reliable genotyping performance for both methods.

No allelic bias was detected in either of the enrichment methods, which showed comparable allele depth ratios to the shotgun data (Table 1, Figure S5). Allele depth ratios across all variant classes and methods were generally close to 0.5, with the exception of transitions. All samples, including both enrichment and shotgun data sets, showed a pattern of elevated cytosine in CT SNPs (and analogous for guanine in AG SNPs).

**TABLE 1:** Summary of allele depth ratios across all biallelic heterozygous sites present in the shotgun data and both enrichment data sets.

Sample	Method	Transition		Transversion			
		AG	CT	AC	AT	CG	GT
BRW001 (*n* = 11,281)	shotgun	**0.475**	**0.533**	0.494	0.501	0.513	0.503
	myBaits	**0.464**	**0.561**	0.495	**0.515**	0.504	0.511
	Twist	**0.472**	**0.541**	0.501	0.507	0.490	0.503
VKP001 (*n* = 9439)	shotgun	**0.467**	**0.544**	0.509	0.506	**0.476**	0.492
	myBaits	**0.452**	**0.579**	0.503	**0.512**	0.502	0.497
	Twist	**0.456**	**0.556**	0.513	0.500	0.486	0.500
DVT014 (*n* = 3331)	shotgun	**0.401**	**0.634**	0.513	0.492	0.510	**0.559**
	myBaits	**0.400**	**0.641**	0.510	0.512	0.518	**0.549**
	Twist	**0.400**	**0.633**	0.520	0.494	0.531	**0.539**
NCP002 (*n* = 72)	shotgun	0.449	0.544	**0.883**	0.438	0.444	**0.700**
	myBaits	0.424	0.589	**0.826**	0.543	**0.692**	0.455
	Twist	**0.396**	0.549	**0.852**	0.462	0.526	0.571

*Note:* An estimated allele depth ratio (est. AD ratio) of 0.5 indicates equal allele counts, a ratio greater than 0.5 indicates a higher count of the first allele, and a ratio less than 0.5 indicates a higher count of the second allele for each variant class. Allele depth ratios differing significantly from the expectation of 0.5 (*p* value < 0.05) are shown in bold, sites remaining significant after Bonferroni correction are additionally coloured (yellow: > 0.5; blue: < 0.5).

## 4 Discussion

We evaluated the performance of two commercially available in‐solution enrichment probe systems, ssRNA‐based myBaits and dsDNA‐based Twist, for their ability to capture target regions of endogenous DNA from ancient crow bones. Both systems effectively enriched ancient DNA with sequencing depth allocated for each enriched library less than the equivalent of 1/40× shotgun sequencing, substantially reducing the sequencing effort otherwise required for degraded samples with low endogenous DNA content (Table S3; mean endogenous DNA = 14.2%, median = 9.6%). Importantly, both kits support custom panel designs, making them adaptable for nonmodel organisms.

### 4.1 On‐Target Rates, Target Enrichment and Fold Enrichment

In our experiments, the myBaits system exhibited higher on‐target rates relative to the total number of sequenced reads, indicating a greater ability to capture targeted regions from the complex pool of endogenous and exogenous DNA in the libraries. Given that raw sequencing reads often contain residual adapter sequences and short fragments that are difficult to fully remove, we focused our comparison on reads mapped to the crow genome. While Twist retained a higher level of endogenous DNA, most of this comprised off‐target alignments, resulting in lower target efficiency. Conversely, the myBaits system consistently achieved higher target efficiency, reflecting greater specificity and capture efficiency. This enhanced specificity may be attributed to a multitude of factors, including the formation of stronger and more stable RNA–DNA hybridisation compared to DNA–DNA heteroduplexes (Chein and Davidson 1978; Lesnik and Freier 1995). Additionally, the shorter probe length of myBaits (60 bp vs. 80 bp for Twist) may also contribute to higher specificity (Suzuki et al. 2007), though shorter probes are generally less effective when targeting highly divergent species, which is not a limitation in this study (Li et al. 2013; Peñalba et al. 2014).

While previous studies have demonstrated that incubation temperature can influence hybridisation stringency and on‐target alignment rates (Cruz‐Dávalos et al. 2017; Paijmans et al. 2016), the incubation temperatures applied in this study were similar between the two probe systems (62°C for Twist and 60°C for myBaits). Nevertheless, direct comparisons between the two enrichment systems are challenging, as DNA melting temperatures are affected by probes, hybridisation reagents, and blockers provided by each manufacturer.

In addition to the lower on‐target alignment rates observed with Twist, we found that both target efficiency and fold enrichment appeared to plateau at approximately 20% and 7×, respectively, across all samples. This pattern hints at a possible inefficiency or saturation in the hybridisation performance of Twist probes under our experimental conditions. This limitation could be related to suboptimal incubation temperature and hybridisation time.

### 4.2 Mitochondrial DNA

Interestingly, we observed a higher proportion of mitochondrial DNA retained as off‐target in the myBaits system compared to Twist, particularly in a poor‐quality sample (NPC002). Overall, the proportion of mitochondrial reads was very low in both designs, especially in Twist, suggesting that co‐capture due to sequence similarity is unlikely. The key difference between the two methods emerged in samples with high mitochondrial levels, such as NCP002, where myBaits retained substantially more mitochondrial reads than Twist. This may reflect an increased affinity of RNA probes in myBaits for short degraded mitochondrial fragments, potentially due to partial sequence similarity and the stronger binding stability of RNA–DNA hybrids compared with DNA–DNA hybrids.

### 4.3 Coverage and GC‐Rich Regions

Due to its higher target efficiency, myBaits also achieved greater mean coverage compared to Twist in the experiments. Both methods displayed similar coverage evenness across target sites, an important factor for avoiding enrichment bias in specific genomic regions. However, the two systems showed notable differences in their efficiency at capturing GC‐rich regions. Twist demonstrated higher efficiency in regions with moderate to high GC content and showed greater tolerance for extreme GC‐rich regions compared with myBaits. Conversely, myBaits performed well in regions with moderate GC content, but showed reduced efficiency in higher GC regions. These results are congruent with previous studies indicating that dsDNA and ssDNA probes outperform RNA‐based probes in GC‐rich regions (Sundararaman et al. 2023; Zhou et al. 2021). However, unlike Zhou et al. (2021), we did not observe significantly better performance of the RNA probes in AT‐rich regions. Therefore, Twist appears better suited for studies targeting species or genomic regions with high GC content.

### 4.4 Detection of Target SNPs

Overall, both enrichment systems recovered substantially more target SNPs than shotgun sequencing, particularly in poor‐quality samples with low endogenous DNA, while requiring significantly less sequencing effort (1.4% vs. up to 80% of a lane per sample). Both kits achieved comparable site detection rates (genotyping success) and genotyping accuracy, with Twist showing slightly higher genotyping accuracy in the more degraded samples (DVT014 and NCP002). This may, however, be due to genotyping issues in low‐quality reads or the failure of shotgun sequencing to sample alternative alleles sufficiently in these degraded samples. Importantly, in decent‐quality samples, both kits achieved high genotype concordance with the shotgun sequencing baseline, with up to 99.1% allelic match for myBaits and 95.9% for Twist, indicating high genotyping accuracy after target enrichment.

### 4.5 Allelic Bias

Both strategies of using probes with both allele types (myBaits) or a third ‘neutral’ allele (Twist) produced consistent and reliable results. No allelic capture bias was detected in either enrichment method, as allele depth ratios were generally consistent with those observed in shotgun sequencing. The allelic imbalance at transition variants, which favoured G and C alleles across both enrichment and shotgun data sets, is unlikely to be the result of GC bias, as no comparable imbalance was observed at transversion variants involving these two alleles. This pattern is likely attributed to postmortem DNA damage. Deamination‐driven C→T (and complementary G→A) transitions are common in aDNA and would typically lead to an excess of A and T reads. However, these lesions may also contribute to genotyping errors, misclassifying homozygous CC (or GG) sites as heterozygous CT (or GA). The apparent excess of C (or G) may actually reflect the true homozygous CC (or GG) genotype, erroneously interpreted as a heterozygote due to damage‐induced misincorporation. This observation highlights the importance of accounting for DNA damage in genotype calling of aDNA samples, for both enrichment and shotgun sequencing methods, especially at transition sites that are prone to miscalls.

Our study has several limitations that remain to be addressed in future studies. A key difference between the two designs is the panel size, which may complicate a direct comparison of enrichment efficiency, as the larger number of probes for more target sites may increase the chance of off‐target binding. We accounted for this by proportionally adjusting the number of off‐target reads in the Twist data set when calculating target efficiency, yet myBaits still consistently outperformed Twist in capture efficiency.

We also note that the input volume of DNA differed slightly between the two methods (7 μL for myBaits and 5 μL for Twist), which may have influenced coverage comparisons. The input volume for myBaits followed the manufacturer's recommendation, whereas the Twist protocol originally recommended using dry pellets, which is not ideal for ancient DNA as pelleting libraries eluted in buffer could lead to additional loss and degradation. To avoid this, we used a reduced input volume for Twist without pelleting. Although this lower input may have contributed to the reduced coverage of targeted SNP sites for Twist, myBaits still achieved higher mean coverage when the comparison was restricted to unique reads with duplicates removed, suggesting that the performance of Twist is unlikely to have been limited by endogenous DNA availability.

Future studies could investigate whether increasing probe tiling in the Twist system would improve on‐target enrichment efficiency. In this experiment, the Twist design used a single double‐stranded DNA probe per target SNP. Increasing probe density by tiling two probes per site to achieve 4× probe coverage or four probes per site to achieve 4× tiling, as implemented in the myBaits design (four single‐stranded RNA probes to get 4× probe coverage and 4× tiling), may enhance on‐target enrichment efficiency and coverage for Twist. Although we accounted for this difference by proportionally adjusting the on‐target read counts to reflect 4× probe coverage, myBaits still demonstrated higher target efficiency. However, this proportional adjustment is a theoretical representation and may not reflect the actual outcome of experimentally increasing probe coverage.

## 5 Conclusion

Overall, our study demonstrates that both custom probe kits substantially improved fold enrichment and target site detection rates compared with shotgun sequencing, while requiring significantly less sequencing effort. Between the two kits, myBaits consistently outperformed Twist in all capture efficiency metrics. Although Twist retained a greater proportion of endogenous DNA, its target efficiency remained low under our experimental conditions, indicating potential for protocol optimisation to reduce off‐target binding. Twist achieved higher coverage in challenging regions with extreme GC content, which could be promising for applications targeting GC‐rich genomic regions.

## Author Contributions

C.Y.G. designed the SNP panel, carried out enrichment experiments, processed the raw data and performed all analyses. L.T. and S.T. conducted collagen extraction and radiocarbon dating. Z.B., T.T. and Z.M.B. assisted with sample provision and identification of crow bones. J.B.W.W. devised the research idea and supervised the overall study. All authors participated in the writing of the final manuscript.

## Conflicts of Interest

The authors declare no conflicts of interest.

## Supporting information


**Data S1:** men70071‐sup‐0001‐FigureS1‐S5‐TableS1‐S5.pdf.

## Data Availability

All sequence data has been deposited at the National Center for Biotechnology Information (NCBI) under BioProject PRJNA989404. Consult Table S1 for accession numbers of individual samples. All code is available at https://github.com/EvoBioWolf/CORVID_aDNAenrichment.
