# Supplementary material for: Performance of Two Custom Probe Kits for In‐Solution Enrichment of Ancient Avian DNA
Source: Mol Ecol Resour. 2025 Nov 10;26(1):e70071. doi: 10.1111/1755-0998.70071 (PMC12627907; doi:10.1111/1755-0998.70071)
Supplement: Supplementary file 1 — Data S1: men70071‐sup‐0001‐FigureS1‐S5‐TableS1‐S5.pdf. [file MEN-26-e70071-s001.pdf]

Supplementary Material

for

Performance of Two Custom Probe Kits for In-solution Enrichment of Ancient Avian DNA

Chyi Yin Gwee, Laura Tassoni, Zlatozar Boev, Teresa Tomek, Zbigniew M. Bochenski, Sahra Talamo, Jochen B.W. Wolf

Table of Contents

Supplementary Text 2

SNP panel design 2

Supplementary Figures 4

Supplementary Figure 1. Comparisons of target efficiency between myBaits and Twist with additional adjustment to on-target and off-target counts for Twist 4

Supplementary Figure 2. Relationship between the performance of myBaits and Twist systems and endogenous DNA 5

Supplementary Figure 3. Coverage distribution 6

Supplementary Figure 4. Coverage distribution 7

Supplementary Figure 5. Allele depth ratios for all samples across the six variant classes 8

Supplementary Tables 9

Supplementary Table 1. Sample information including the NCBI accession number . 9

Supplementary Table 2. Radiocarbon dates 11

Supplementary Table 3. Summary statistics of each sample shotgun sequence 13

Supplementary Table 4. Summary statistics of the mean and standard deviation 15

Supplementary Table 5. Total number of reads aligned to the target 104K SNP sites including duplicates and with duplicates removed 17

Literature cited 18

Supplementary Text

SNP panel design

The 104K SNP panel includes 38,498 divergent SNPs and 65,271 putatively neutral SNPs identified across the *Corvus corone* species complex in previous studies (Poelstra et al., 2014; Vijay et al., 2016). The divergent SNPs represent highly differentiated genomic regions (F_{ST} in the 99.9th percentile and d_{XY} in the 99.95 percentile) between populations within three hybrid zones: (1) the all-black German and gray-coated Polish/ Swedish populations in the European hybrid zone, (2) the gray-coated and all-black Russian populations in the Siberian hybrid zone, and (3) the gray-coated Russian and pied Central Asian populations in the Asian hybrid zone (Knief et al., 2019; Vijay et al., 2016).

The 65,271 putatively neutral SNPs were selected from intronic, intergenic, and fourfold-degenerate sites across seven discovery populations. Each population was represented by 15 Spanish (cor1), 15 German (cor2), 15 Polish/ Swedish (cnx3), five West Siberian (ori1), three Central Siberian (ori2), six East Siberian (ori3), and two Central Asian (pec1) crows (Vijay et al., 2016). These populations were chosen due to their distinct population structures. While hooded crows are distributed widely across central, eastern, northern and southern Europe, they exhibit genomic homogeneity, thus Polish and Swedish crows were deemed sufficiently representative of the gray-coated hooded crow. Despite the under-representation of the Siberian and Asian populations, the European populations (cor1, cor2, and cnx3) were equally well-represented.

A total of 2,750,278 intronic, 8,747,636 intergenic, and 47,453 fourfold-degenerate SNPs were identified across the seven populations. To obtain unlinked SNPs, 1 SNP per 10,000 bp was sampled from the intronic and intergenic sites, and 1 SNP per 1,000 bp was sampled from the fourfold-degenerate sites of each population separately. This pruning process reduces the available SNPs to 113,800 intronic, 347,839 intergenic, and 22,443 fourfold-degenerate SNPs. All 22,443 fourfold-degenerate SNPs were included in the panel, while the intronic and intergenic SNPs were further down-sampled by randomly selecting 17,250 intronic and 54,900 intergenic SNPs from the pooled SNPs of all populations. From this pool of randomly selected SNPs, linkage pruning was conducted as before, resulting in 11,286 intronic and 32,732

intergenic SNPs. After removing non-biallelic SNPs, a total of 65,271 neutral SNPs were retained in the final panel. To assess the representativeness of the selected neutral SNP panel, we conducted principal component analysis (PCA) on all 11 million neutral SNPs and the 65,271 selected SNPs. The results demonstrated similar population structure, validating the selection process.

The 232K SNP panel used in the Twist design includes all 104K SNPs from the myBaits panel, along with an additional 128,246 putatively neutral SNPs. These additional SNPs were derived in part from the inclusion of the Iraqi population, represented by five individuals (Gwee et al, 2025), and through the use of an outgroup ascertained sampling approach. SNP sampling for the Iraqi population followed a similar approach to the one described previously. After pruning for linkage disequilibrium, 20,909 intronic, 59,161 intergenic, and 4,475 fourfold-degenerate sites were retained. To scale to 1/7th of the previous neutral SNP panel design, 1,614 intronic and 4,714 intergenic SNPs were randomly selected. Following the removal of non-biallelic SNPs and SNPs overlapping with the existing panel, a total of 8,217 neutral SNPs were added to the Twist panel.

The 104K SNP panel of myBaits consists of neutral SNPs from seven discovery populations (subpanels 1-7) and the 232K SNP panel of Twist consists neutral SNPs from eight discovery populations (subpanels 1-10). The complete neutral SNP panel consists of 10 subpanels, designed using a strategy similar to the 13 panels of the Affymetrix Human Origins array (Patterson et al., 2012):

- Subpanels 1-8 represent heterozygous sites from each of the eight discovery populations, totaling 73,488 SNPs. While the Affymetrix Human Origins arrays discover SNPs from one genome representing each population, we discover SNPs from several individuals of each population.
- Subpanel 9 was designed with an outgroup-ascertained approach, where the outgroup American crow (*C. brachyrhynchos*) and any of the eight discovery populations are heterozygous, resulting in 72,688 unlinked transversion SNPs.
- Subpanel 10 includes 47,341 additional unlinked transversion SNPs shared across all eight discovery populations.

Figures

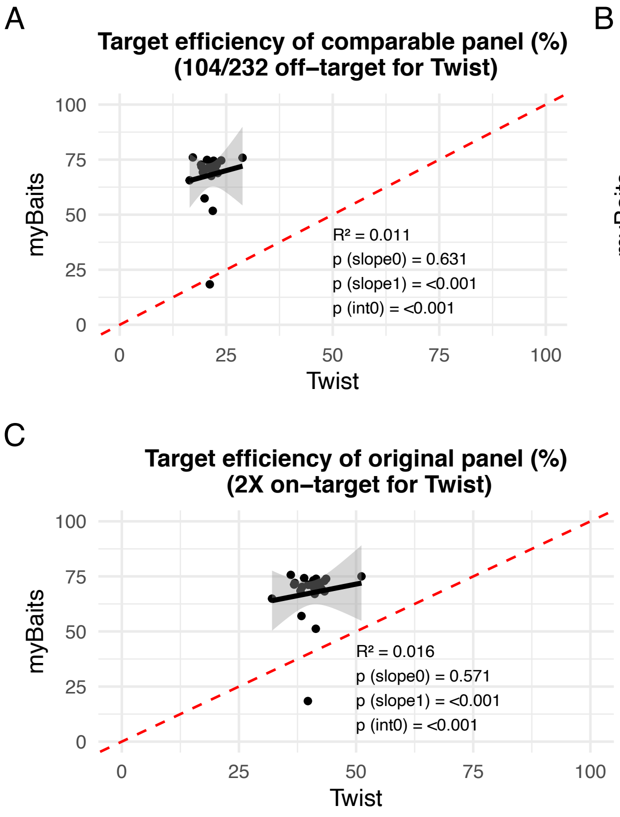

Supplementary Figure 1. Comparisons of target efficiency between myBaits and Twist with additional adjustment to on-target and off-target counts for Twist. Each dot represents one of 23 samples to which both strategies were applied for plots **A-D** and **G**. The red dashed line indicates the expected 1:1 relationship under equal performance, the black solid line shows the observed linear regression, and the grey shaded area depicts the 95% confidence interval of the regression. Correlation value (R^2), the significance of the linear relationship (p(slope0)), the significance of non-proportional scaling (p(slope(1)), and the significance of a non-zero intercept (p(int0)) are reported in each plot. (**A**) For the comparable panel, the off-target count for Twist was proportionally reduced (104/232) to account for the potential effect of higher off-target binding due to the larger panel size. (**B**) For the comparable panel, the off-target count for Twist was proportionally reduced as in (**A**) and the on-target count was doubled to adjust for the 4X probe coverage in myBaits. (**C**) For the original panel, the on-target count for Twist was doubled to match the probe coverage in myBaits.

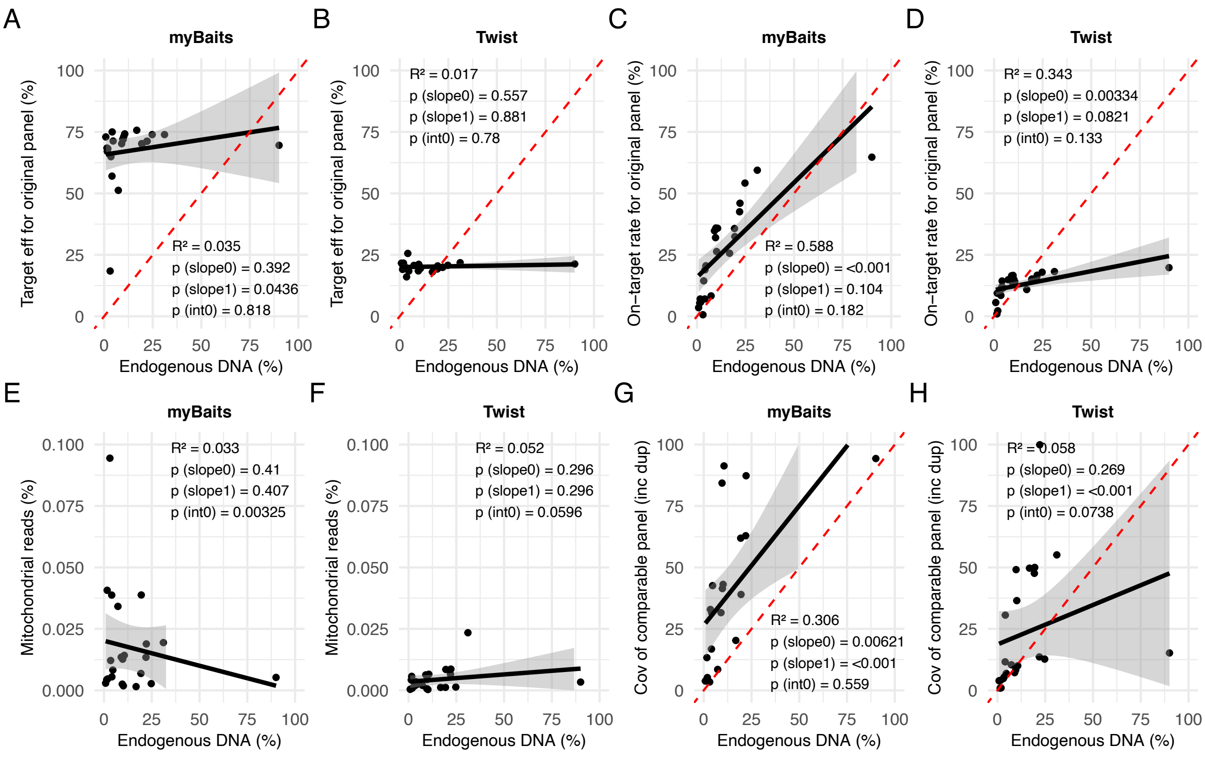

Supplementary Figure 2. Relationship between the performance of myBaits and Twist capture systems and endogenous DNA content, the latter quantified by shallow shotgun sequencing. Each dot represents one of 23 samples to which both strategies. The red dashed line indicates the expected 1:1 relationship under equal performance, the black solid line shows the observed linear regression, and the grey shaded area depicts the 95% confidence interval of the regression. Correlation value (R^2), the significance of the linear relationship (p(slope0)), the significance of non-proportional scaling (p(slope(1)), and the significance of a non-zero intercept (p(int0)) are reported in each plot. (**A**) Target efficiency of myBaits using the original panel (104K SNPs with 121 bp probes) against endogenous DNA content. (**B**) Target efficiency of Twist using the original panel (232K SNPs with 80 bp probes) against endogenous DNA content. (**C**) On-target rate of myBaits using the original panel (104K SNPs with 121 bp probes) against endogenous DNA content. (**D**) On-target rate of Twist based on the original panel (232K SNPs with 80 bp probes) against endogenous DNA content. (**E**) Proportion of mitochondrial reads in myBaits-enriched libraries against endogenous DNA content. (**F**) Proportion of mitochondrial reads in Twist-enriched libraries against endogenous DNA content. (**G**) Coverage of the comparable panel (104K SNPs with 80 bp probes) using myBaits against endogenous DNA content. (**H**) Coverage of the comparable panel (104K SNPs with 80 bp probes) using Twist against endogenous DNA content.

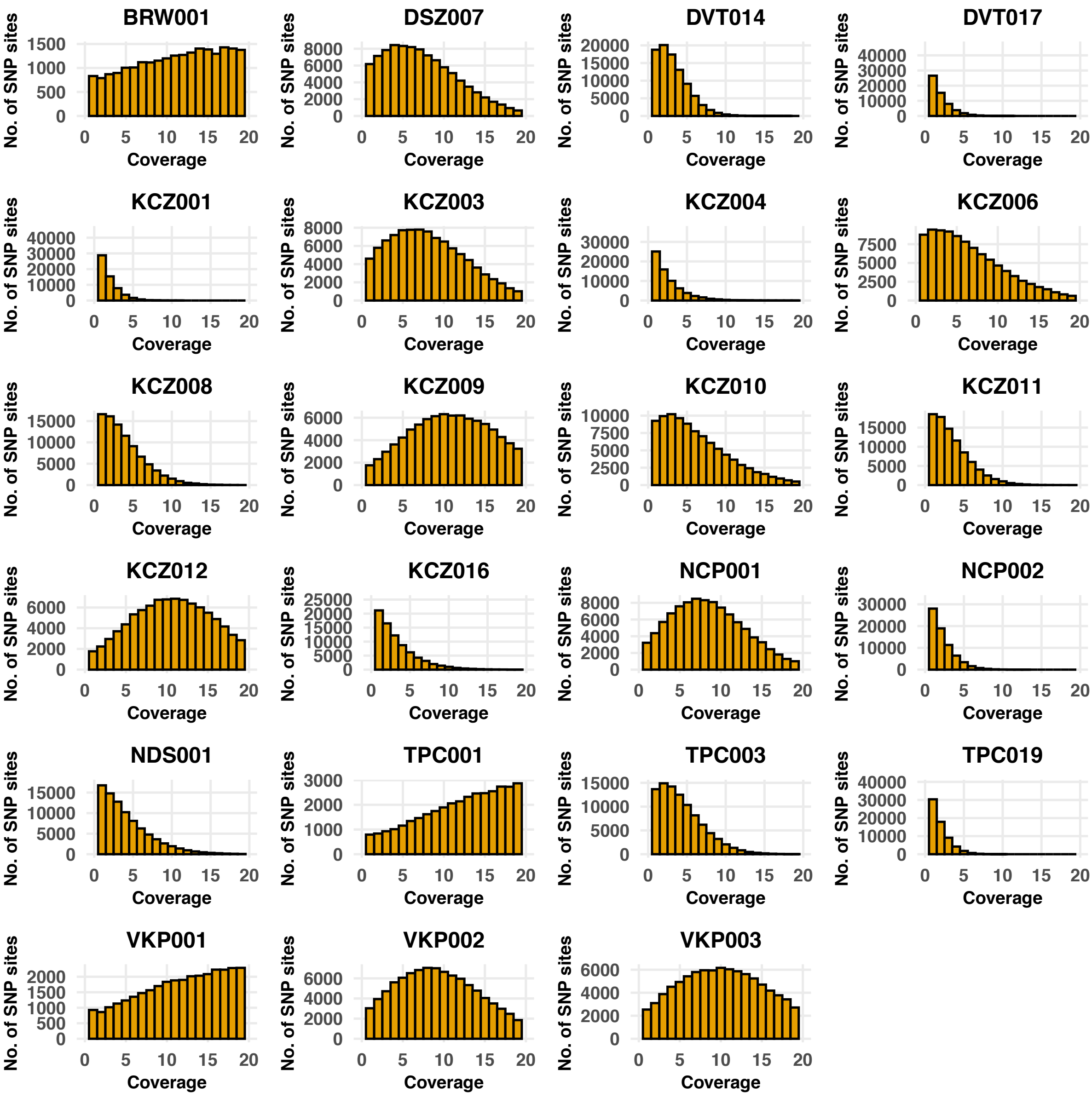

Supplementary Figure 3. Coverage distribution of each of the 23 samples enriched with myBaits.

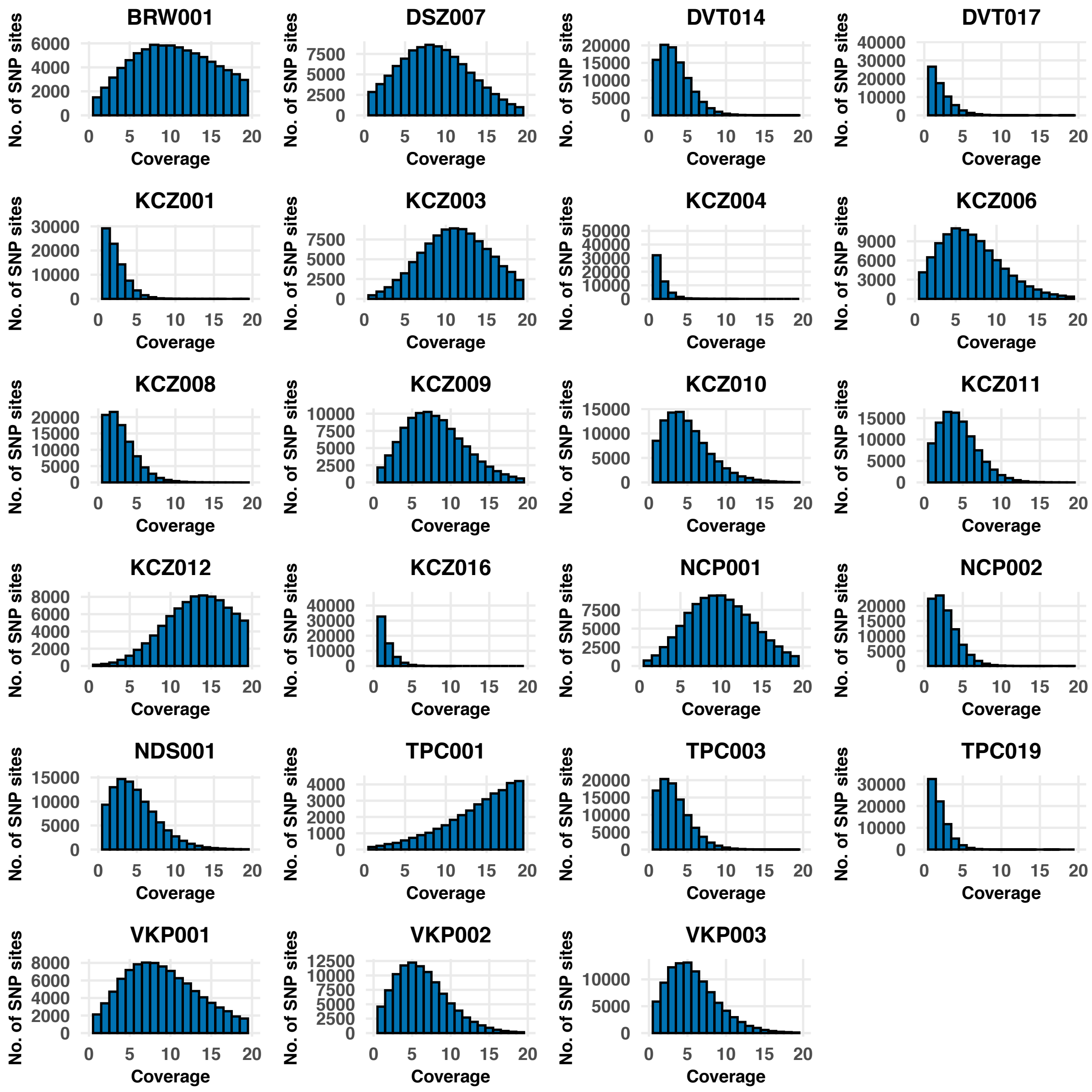

Supplementary Figure 4. Coverage distribution of each of the 23 samples enriched with Twist

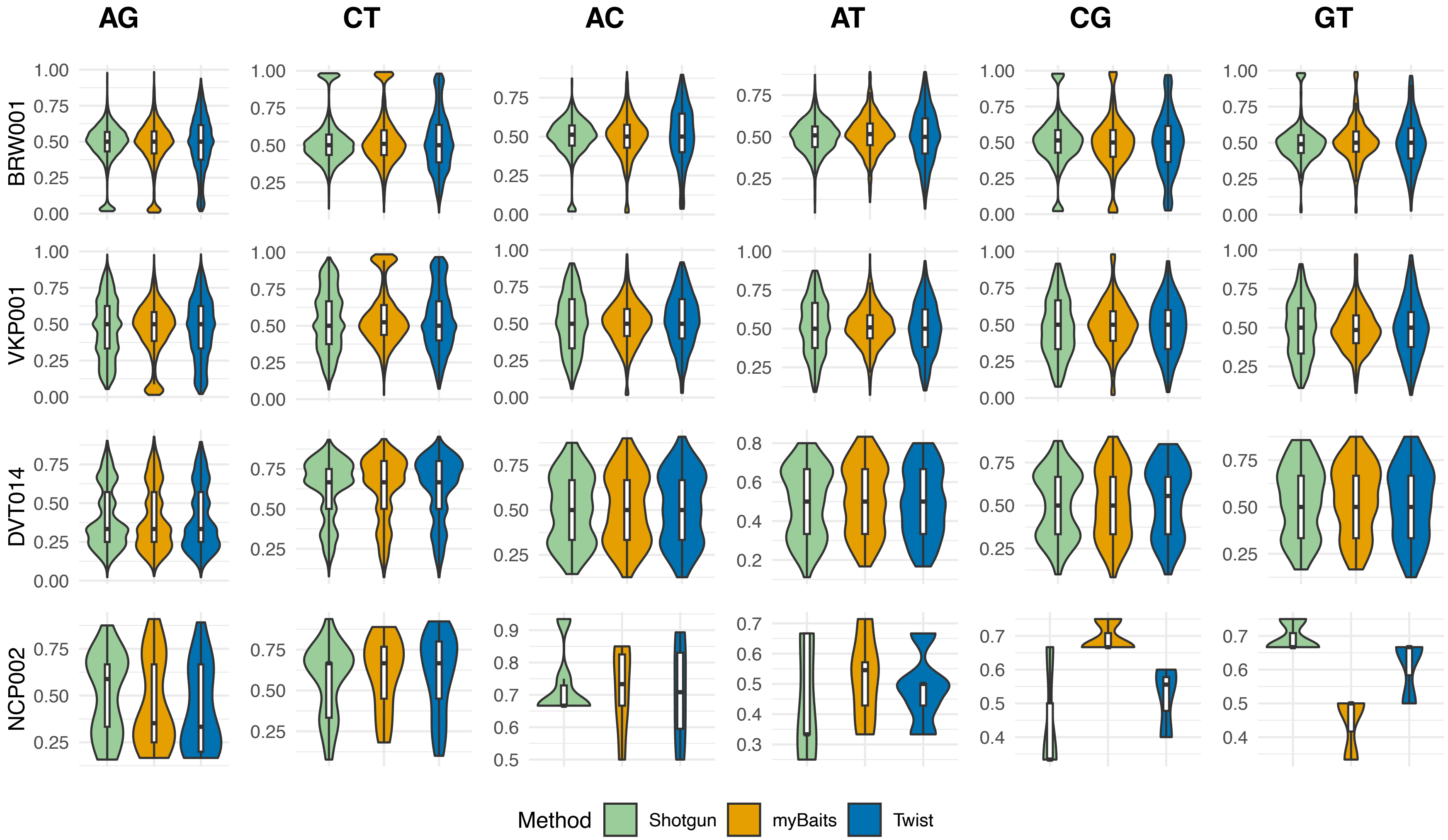

Supplementary Figure 5. Allele depth ratios for all samples across the six variant classes. Note that the sample NCP002 retained only 67 variant sites after filtering, leading to a skewed distribution compared to the other samples.

Supplementary Tables

Supplementary Table 1. Sample information including the NCBI accession number of enriched sequences and whole genome sequences of four selected samples.

Sample ID	NCBI SAMN No.	NCBI SRR No. (Twist)	NCBI SRR No. (myBaits)	NCBI SRR No. (WGS)
BRW001	SAMN38669687	SRR27127887	SRR30589989	SRR30589965, SRR30589953, SRR30589941, SRR27127872, SRR27127871, SRR27127873
DSZ007	SAMN36085508	SRR25080804	SRR30589978	
DVT014	SAMN36085511	SRR25080800	SRR30589967	SRR30589952, SRR30589940, SRR30589964, SRR27127868, SRR27127870, SRR27127869
DVT017	SAMN36085512	SRR25080799	SRR30589945	
KCZ001	SAMN38669693	SRR27127836	SRR30590007	
KCZ003	SAMN36085513	SRR25080798	SRR30590006	
KCZ004	SAMN38669694	SRR27127908	SRR30590005	
KCZ006	SAMN38669695	SRR27127907	SRR30590004	
KCZ008	SAMN38669696	SRR27127906	SRR30590003	
KCZ009	SAMN38669697	SRR27127905	SRR30590002	
KCZ010	SAMN38669698	SRR27127904	SRR30590001	
KCZ011	SAMN38669699	SRR27127903	SRR30589999	
KCZ012	SAMN36085514	SRR25080797	SRR30589998	
KCZ016	SAMN38669700	SRR27127902	SRR30589997	
NCP001	SAMN36085515	SRR25080796	SRR30589992	
NCP002	SAMN38669705	SRR27127896	SRR30589991	SRR30589958, SRR30589933, SRR30589946, SRR27127850, SRR27127849, SRR27127848

NDS001	SAMN38669706	SRR27127895	SRR30589990	
TPC001	SAMN36085509	SRR25080802	SRR30589977	
TPC003	SAMN38669716	SRR27127884	SRR30589976	
TPC019	SAMN36085510	SRR25080801	SRR30589975	
VKP001	SAMN38669718	SRR27127882	SRR30589973	SRR30589955, SRR30589931, SRR30589943, SRR27127842, SRR27127844, SRR27127841
VKP002	SAMN38669719	SRR27127881	SRR30589972	
VKP003	SAMN38669720	SRR27127880	SRR30589971	

Supplementary Table 2. Radiocarbon dates of 22 samples by Accelerator Mass Spectrometry (AMS). One sample, TPC019, was excluded from radiocarbon dating due to insufficient material.

Sample ID	Site Name	BRAVHO Code	samples taken	collagen		Graphite code	N %	C %	C:N	AMS Lab Code	^{14}C Age	1s Err	$d^{13}C$	$F^{14}C$	±(%)	Calibrated Ages	
			[mg]	[mg]	[%]											68,30%	95,40%
BRW001	Birow	BRA-6385	61,3	8,5	13,9	B.AGE-518	15,18	43,35	2,86	MAMS-63412	1,904	19	-16,6	0,789	0,186	cal AD 85-203	cal AD 77-210
DSZ007	Deszczowa	BRA-6376	125,6	20,4	16,2	B.AGE-509	15,38	44,50	2,89	MAMS-63403	332	18	-21,5	0,959	0,212	cal AD 1505-1633	cal AD 1490-1638
DVT014	Devetashka Cave	BRA-6349	90,3	13,0	14,4	B.AGE-462	16,24	46,69	2,87	MAMS-62586	13,319	39	-17,6	0,190	0,087	cal BC 14035-13884	cal BC 14103-13810
DVT017	Devetashka Cave	BRA-6351	108,3	12,5	11,5	B.AGE-464	15,54	44,85	2,89	MAMS-62588	13,732	37	-18,0	0,181	0,077	cal BC 14623-14465	cal BC 14697-14398
KCZ001	Krucza Skala	BRA-6354	273,9	26,4	9,6	B.AGE-467	16,35	47,09	2,88	MAMS-62591	717	18	-18,0	0,915	0,202	cal AD 1278-1290	cal AD 1273-1375
KCZ003	Krucza Skala	BRA-6357	130,6	10,0	7,7	B.AGE-470	15,66	44,31	2,83	MAMS-62594	282	18	-18,8	0,965	0,212	cal AD 1529-1652	cal AD 1524-1660
KCZ004	Krucza Skala	BRA-6363	170,9	13,3	7,8	B.AGE-476	16,01	46,15	2,88	MAMS-62600	673	19	-17,9	0,920	0,214	cal AD 1288-1380	cal AD 1282-1387
KCZ006	Krucza Skala	BRA-6364	143,5	20,7	14,4	B.AGE-477	16,26	46,05	2,83	MAMS-62601	427	18	-22,1	0,948	0,211	cal AD 1444-1467	cal AD 1438-1478
KCZ008	Krucza Skala	BRA-6358	183	28,5	15,6	B.AGE-471	16,36	46,36	2,83	MAMS-62595	350	18	-18,3	0,957	0,216	cal AD 1492-1628	cal AD 1476-1634
KCZ009	Krucza Skala	BRA-6360	139,5	21,3	15,3	B.AGE-473	16,29	46,40	2,85	MAMS-62597	922	18	-23,3	0,892	0,199	cal AD 1049-1165	cal AD 1041-1202
KCZ010	Krucza Skala	BRA-6359	170	28,8	16,9	B.AGE-472	16,27	46,30	2,85	MAMS-62596	270	18	-18,5	0,967	0,219	cal AD 1533-1658	cal AD 1527-1794
KCZ011	Krucza Skala	BRA-6361	194,3	25,2	13,0	B.AGE-474	16,43	46,84	2,85	MAMS-62598	647	18	-20,2	0,923	0,202	cal AD 1300-1387	cal AD 1293-1392
KCZ012	Krucza Skala	BRA-6362	192	31,2	16,3	B.AGE-475	16,59	47,28	2,85	MAMS-62599	773	18	-21,0	0,908	0,205	cal AD 1233-1277	cal AD 1227-1278
KCZ016	Krucza Skala	BRA-6365	130	8,1	6,2	B.AGE-478	15,16	44,20	2,92	MAMS-62602	472	18	-19,0	0,943	0,205	cal AD 1431-1445	cal AD 1424-1451
NCP001	Nicopolis-ad-Istrum	BRA-6387	174,8	25,9	14,8	B.AGE-487	16,39	46,50	2,84	MAMS-62611	1,639	20	-19,4	0,815	0,203	cal AD 412-529	cal AD 405-536
NCP002	Nicopolis-ad-Istrum	BRA-6370	89,2	14,3	16,0	B.AGE-483	15,90	45,12	2,84	MAMS-62607	1,872	21	-19,4	0,792	0,201	cal AD 130-215	cal AD 126-231

NDS001	Niedostepna Cave	BRA-6375	150,5	5,7	3,8	B.AGE-508	15,33	43,44	2,83	MAMS-63402	370	19	-26,2	0,955	0,220	cal AD 1469-1618	cal AD 1455-1629
TPC001	Topchii	BRA-6377	137,4	17,6	12,8	B.AGE-510	15,59	45,11	2,89	MAMS-63404	197	18	-21,1	0,976	0,220	cal AD 1661-1800	cal AD 1657-1950
TPC003	Topchii	BRA-6378	125,2	21,9	17,5	B.AGE-511	16,88	47,92	2,84	MAMS-63405	164	18	-18,1	0,980	0,218	cal AD 1674-1942	cal AD 1666-1950
VKP001	Veliki Preslav	BRA-6382	96,3	13,8	14,3	B.AGE-515	15,29	44,59	2,92	MAMS-63409	883	18	-21,1	0,896	0,206	cal AD 1163-1210	cal AD 1053-1219
VKP002	Veliki Preslav	BRA-6383	73,3	10,7	14,6	B.AGE-516	15,48	44,00	2,84	MAMS-63410	1,138	18	-18,7	0,868	0,200	cal AD 886-972	cal AD 774-987
VKP003	Veliki Preslav	BRA-6384	91,6	14,0	15,3	B.AGE-517	15,35	43,99	2,87	MAMS-63411	1,159	18	-16,9	0,866	0,198	cal AD 776-950	cal AD 772-973
TPC019	Topchii	NA	NA	NA	NA	NA	NA	NA	NA	NA	NA	NA	NA	NA	NA	NA	NA

Supplementary Table 3. Summary statistics of each sample shotgun sequenced to estimate the expected genomic coverage of the library. The table also includes information of the DNA concentration of non-enriched indexed libraries and the amount of endogenous DNA per sample. All 23 samples were sequenced shallowly at MPI-EVA, with four samples (in bold) sequenced more deeply with Novogene. Note that the informative sequence count was calculated for the entire 40uL of the library, of which 7uL and 5uL were used for myBaits and Twist, respectively. The expected total genomic coverage was calculated with the assumption that the estimated sequence content is evenly distributed across a 1.12 billion bp genomic region.

Sample Name	DNA library conc (ng/uL)	Endogenous DNA (%)	Deduplicated mapped reads	Total reads	Mean read length	qPCR raw molecule count	Informative sequence content of the entire 40uL library (Mbp)	Expected total genomic coverage
**BRW001**	203.3	90.05	2.60E+06	4.68E+06	48.1	5.47E+10	2.37E+06	2113.0
DSZ007	273.9	10.13	3.15E+05	4.95E+06	44.6	5.02E+09	2.27E+04	20.2
**DVT014**	180.1	19.51	3.17E+05	3.41E+06	41.7	1.17E+09	9.52E+03	8.5
DVT017	140.9	16.79	2.55E+05	3.59E+06	40.0	9.05E+08	6.07E+03	5.4
KCZ001	214.9	0.86	1.81E+04	3.63E+06	43.6	1.57E+10	5.91E+03	5.3
KCZ003	188.5	9.76	2.24E+05	3.58E+06	46.0	5.38E+09	2.42E+04	21.6
KCZ004	241.1	1.45	3.19E+04	3.85E+06	41.7	5.80E+10	3.51E+04	31.4
KCZ006	274.6	4.04	1.46E+05	5.45E+06	47.9	1.61E+10	3.12E+04	27.9
KCZ008	167.6	1.66	5.31E+04	4.52E+06	45.9	1.75E+10	1.33E+04	11.9
KCZ009	231.9	21.96	9.27E+05	6.93E+06	48.2	2.95E+09	3.12E+04	27.9
KCZ010	247.1	4.62	2.10E+05	6.33E+06	46.9	1.13E+10	2.45E+04	21.8
KCZ011	203.6	7.29	2.63E+05	5.68E+06	44.3	3.74E+09	1.21E+04	10.8
KCZ012	246	22.16	6.32E+05	4.64E+06	46.3	3.03E+09	3.11E+04	27.8

KCZ016	167.5	1.90	5.25E+04	5.09E+06	44.3	5.02E+10	4.22E+04	37.7
NCP001	198.3	19.33	3.48E+05	3.14E+06	46.8	2.86E+09	2.59E+04	23.1
**NCP002**	299	3.09	9.06E+04	4.45E+06	46.3	5.49E+09	7.84E+03	7.0
NDS001	148	9.02	1.42E+05	2.62E+06	44.2	6.70E+09	2.67E+04	23.8
TPC001	241.9	31.08	1.06E+06	5.90E+06	46.6	7.33E+09	1.06E+05	94.7
TPC003	193.7	3.49	1.03E+05	4.51E+06	45.8	1.00E+10	1.60E+04	14.3
TPC019	225.8	4.13	1.31E+05	6.62E+06	37.7	4.31E+09	6.72E+03	6.0
**VKP001**	166.2	24.73	5.29E+05	3.58E+06	47.6	1.41E+10	1.66E+05	148.2
VKP002	156.6	10.61	4.89E+05	7.63E+06	48.1	7.94E+09	4.06E+04	36.2
VKP003	147.7	9.57	6.46E+05	1.12E+07	49.1	7.82E+09	3.67E+04	32.8

Supplementary Table 4. Summary statistics of the mean and standard deviation for 23 samples enriched by myBaits and Twist separately. Statistics are reported including duplicates (inc. dup) and with duplicates removed (dedup), where applicable. The comparable panel refers to the 104K SNPs with 80 bp probes overlapping in both enrichment designs, while the original panel refers to the 104K SNPs with 121 bp probes for myBaits and 232K SNPs with 80 bp probes for Twist. Expected genomic coverage (cov) based on input DNA was calculated using the informative sequence content for each sample (see **Supplementary Table 3**), assuming uniform distribution across a 1.12 Gb genome. Coverages were calculated using number (nr.) of reads mapped to the respective target regions (e.g. comparable panel or SNP sites), relative to the total target length.

Mean / St. dev	Mean (inc. dup)		Mean (dedup)		St. dev (inc.dup)		St. dev (dedup)	
**Methods**	**mybaits**	**Twist**	**mybaits**	**Twist**	**mybaits**	**Twist**	**mybaits**	**Twist**
Nr. raw reads	32,630,136	68,039,877	NA	NA	17,566,492	66,112,343	NA	NA
Nr. of mapped reads	12,173,102	47,352,642	2,439,191	9,125,949	10,231,776	51,582,385	2,231,756	6,069,574
Enogenous DNA captured (%)	37.83	63.64	NA	NA	25.58	23.29	NA	NA
Nr. of mtDNA reads	1,593	2,857	669	1,623	1,678	5,806	717	3,159
mtDNA among mapped reads (%)	0.02	0.00	0.03	0.01	0.02	0.00	0.03	0.01
Mapped read length (bp)	53.76	54.80	NA	NA	5.49	5.48	NA	NA
GC of mapped reads	NA	NA	42.96	44.30	NA	NA	0.82	1.22
C to T subst on 1st bp (comparable panel)	NA	NA	17.45	16.66	NA	NA	12.67	12.16
Nr. of reads aligned to original panel	8,628,490	9,578,079	1,385,043	2,119,659	7,644,965	10,270,328	1,494,214	1,682,313
Nr. of reads aligned to comparable panel	8,589,121	4,004,496	1,375,624	906,920	7,603,548	4,306,295	1,482,453	742,529
On-target rate (original panel)	26.83	12.85	54.15	22.09	18.76	4.90	12.97	4.89
On-target rate (comparable panel)	26.90	5.88	54.40	10.79	18.87	2.36	13.05	2.74
Target efficiency (original panel)	67.47	20.18	54.15	22.09	12.08	1.80	12.97	4.89
Target efficiency (comparable panel)	68.07	9.56	54.40	10.79	12.23	1.09	13.05	2.74
Expected genomic cov based on input	16.78	11.99	NA	NA	60.99	43.57	NA	NA
Cov of comparable panel	49.41	23.24	7.57	5.27	45.22	24.94	8.84	4.40

Cov of 104K SNP sites	61.40	31.21	8.85	6.70	56.06	33.60	10.32	5.52
SNPs with at least 1X cov (%)	85.70	89.22	85.70	89.22	0.15	0.15	14.56	15.14
SNPs with at least 2X cov (%)	82.60	83.89	73.63	77.48	0.17	0.21	24.33	25.91
SNPs with at least 3X cov (%)	79.64	79.07	63.81	66.79	0.19	0.24	30.06	31.83
SNPs with at least 4X cov (%)	76.91	74.64	55.89	57.51	0.22	0.26	33.16	34.77
SNPs with at least 5X cov (%)	74.37	70.48	49.45	49.69	0.23	0.28	34.57	35.84

Supplementary Table 5. Total number of reads aligned to the target 104K SNP sites including duplicates (inc. dup) and with duplicates removed (dedup). Although the mean total raw reads were higher in Twist (see Supplementary Table 4), the number of reads aligned to SNP sites including duplicates was approximately three times higher in myBaits. After duplicate removal, the number of unique reads aligned to SNP sites was similar between the two methods, likely due to a higher proportion of PCR duplicates in the myBaits libraries being removed during processing.

	**Nr. of reads aligned to 104K SNP sites (inc. dup)**			**Nr. of reads aligned to 104K SNP sites (dedup)**		
	**myBaits**	**twist**	**ratio**	**mybaits**	**twist**	**ratio**
	10,876,959	1,861,707	5.84	4,151,023	1,265,870	3.28
	5,063,832	4,611,524	1.10	706,659	889,931	0.79
	4,714,446	6,467,974	0.73	287,928	334,871	0.86
	2,438,107	6,411,339	0.38	113,307	147,660	0.77
	452,185	492,303	0.92	108,519	178,996	0.61
	4,806,347	6,089,689	0.79	795,837	1,180,821	0.67
	450,907	112,955	3.99	168,853	79,688	2.12
	1,927,030	1,415,031	1.36	614,531	680,402	0.90
	1,551,407	503,848	3.08	330,649	271,447	1.22
	7,311,699	1,687,102	4.33	1,174,319	803,245	1.46
	5,013,462	869,195	5.77	586,789	483,300	1.21
	1,015,826	1,327,074	0.77	295,788	438,749	0.67
	10,316,400	12,590,081	0.82	1,128,118	1,494,278	0.75
	614,736	126,648	4.85	250,895	92,378	2.72
	7,297,348	5,991,083	1.22	843,777	1,033,377	0.82
	394,587	603,890	0.65	155,696	251,015	0.62
	3,759,648	917,920	4.10	348,626	469,064	0.74
	17,825,327	6,874,542	2.59	2,430,349	2,319,965	1.05
	3,894,600	672,175	5.79	389,537	318,603	1.22
	3,764,356	3,838,921	0.98	123,258	152,481	0.81
	19,101,978	1,558,826	12.25	2,768,293	965,821	2.87
	10,656,806	1,257,170	8.48	960,528	622,630	1.54
	9,821,417	1,035,350	9.49	1,096,756	560,363	1.96
**Mean**	5,785,627	2,926,798	3.49	862,175	653,694	1.29
**Median**	4,714,446	1,415,031	2.59	586,789	483,300	0.90
**SD**	5,037,076	3,034,253	3.12	953,703	520,512	0.75

Literature cited

Knief, U., Bossu, C. M., Saino, N., Hansson, B., Poelstra, J., Vijay, N., Weissensteiner, M., & Wolf, J. B. W. (2019). Epistatic mutations under divergent selection govern phenotypic variation in the crow hybrid zone. *Nature Ecology & Evolution*, *3*(4), 570–576. https://doi.org/10.1038/s41559-019-0847-9

Patterson, N. J., Moorjani, P., Luo, Y., Mallick, S., Rohland, N., Zhan, Y., Genschoreck, T., Webster, T., & Reich, D. (2012). Ancient admixture in human history. *Genetics*, *192*, 1065–1093. https://doi.org/10.1534/genetics.112.145037

Poelstra, J. W., Vijay, N., Bossu, C. M., Lantz, H., Ryll, B., Müller, I., Baglione, V., Unneberg, P., Wikelski, M., Grabherr, M. G., & Wolf, J. B. W. (2014). The genomic landscape underlying phenotypic integrity in the face of gene flow in crows. *Science*, *344*(6190), 1410–1414. https://doi.org/10.1126/science.1253226

Vijay, N., Bossu, C. M., Poelstra, J. W., Weissensteiner, M. H., Suh, A., Kryukov, A. P., & Wolf, J. B. W. (2016). Evolution of heterogeneous genome differentiation across multiple contact zones in a crow species complex. *Nature Communications*, *7*, 13195. https://doi.org/10.1038/ncomms13195
